# Localized Modeling of Biochemical and Flow Interactions during Cancer Cell Adhesion

**DOI:** 10.1371/journal.pone.0136926

**Published:** 2015-09-14

**Authors:** Julie Behr, Byron Gaskin, Changliang Fu, Cheng Dong, Robert Kunz

**Affiliations:** 1 Department of Biomedical Engineering, Pennsylvania State University, University Park, Pennsylvania, United States of America; 2 Applied Research Laboratory, Pennsylvania State University, University Park, Pennsylvania, United States of America; A*STAR Bioinformatics Institute, SINGAPORE

## Abstract

This work focuses on one component of a larger research effort to develop a simulation tool to model populations of flowing cells. Specifically, in this study a local model of the biochemical interactions between circulating melanoma tumor cells (TC) and substrate adherent polymorphonuclear neutrophils (PMN) is developed. This model provides realistic three-dimensional distributions of bond formation and attendant attraction and repulsion forces that are consistent with the time dependent Computational Fluid Dynamics (CFD) framework of the full system model which accounts local pressure, shear and repulsion forces. The resulting full dynamics model enables exploration of TC adhesion to adherent PMNs, which is a known participating mechanism in melanoma cell metastasis. The model defines the adhesion molecules present on the TC and PMN cell surfaces, and calculates their interactions as the melanoma cell flows past the PMN. Biochemical rates of reactions between individual molecules are determined based on their local properties. The melanoma cell in the model expresses ICAM-1 molecules on its surface, and the PMN expresses the *β*-2 integrins LFA-1 and Mac-1. In this work the PMN is fixed to the substrate and is assumed fully rigid and of a prescribed shear-rate dependent shape obtained from micro-PIV experiments. The melanoma cell is transported with full six-degrees-of-freedom dynamics. Adhesion models, which represent the ability of molecules to bond and adhere the cells to each other, and repulsion models, which represent the various physical mechanisms of cellular repulsion, are incorporated with the CFD solver. All models are general enough to allow for future extensions, including arbitrary adhesion molecule types, and the ability to redefine the values of parameters to represent various cell types. The model presented in this study will be part of a clinical tool for development of personalized medical treatment programs.

## Introduction

**Fig 1:**
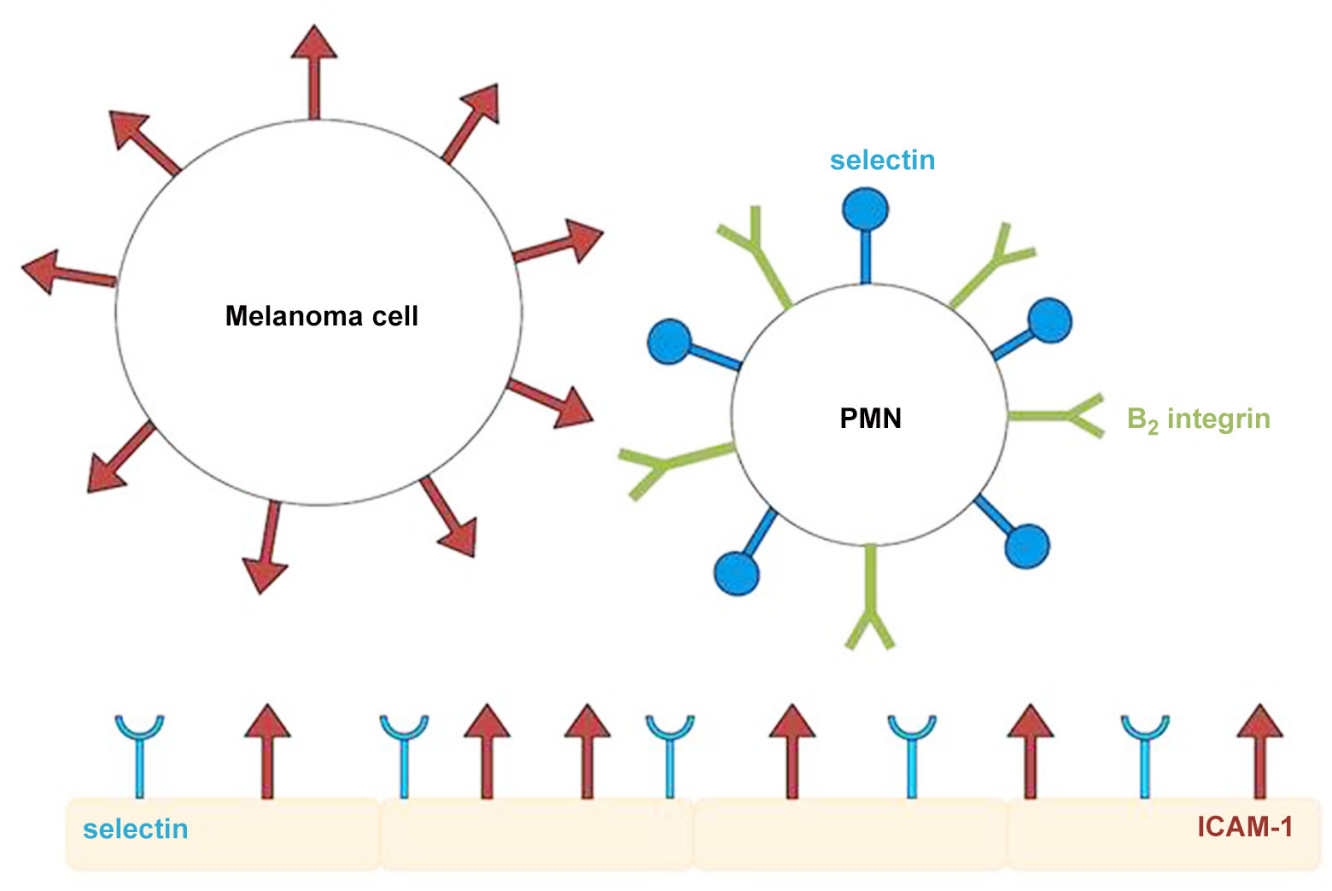
Cartoon of adhesion molecule expression. *β*-2 integrins expressed on the PMN can interact with ICAM-1 expressed on both the melanoma cell and endothelial cells. The PMN and endothelium can also interact through selectins.

Polymorphonuclear neutrophils (PMNs) comprise 50–70% of all circulating white blood cells, or leukocytes [1]. Studies have also shown that inflammatory signals have enhanced the ability of circulating melanoma tumor cells to extravasate [2]. Under some circumstances, PMNs will actually enhance metastatic capabilities, where in most cases they are cytotoxic to tumor cells [3]. The correlation between inflammatory signals and increased melanoma cell metastasis implies that the circulating melanoma cells are able to take advantage of the immune system and use white blood cells to assist their extravasation mechanisms. In a study by Dong, et al, it was determined that, under flow conditions and in the absence of PMNs, metastatic melanoma cells were no more likely than non-metastatic melanocytes to bind to the endothelium [1]. With the addition of PMN, melanoma extravasation increased significantly. This process was dependent on both the molecular interactions between the melanoma cells with the PMNs, and the PMNs with the endothelial cells. Both melanoma cells and endothelial cells express ICAM-1 (intercellular adhesion molecule) on their surfaces. PMNs express *β*-2 integrins, including Mac-1 and LFA-1. Fig 1 shows a simplified representation of the adhesion molecule expressions. Interactions between LFA-1 and ICAM-1 aid in the initial capture of a white blood cell to the endothelium, and interactions between Mac-1 and ICAM-1 aid in stabilizing the adhesion even in shear-flow conditions. Blocking ICAM-1 expression on the melanoma cells and separately on the endothelial cells both resulted in a significant decrease in the number of melanoma cell extravasations.

In recent years, computational models have matured to where they can be used to explore the complex multi-scale (molecular-cell scale) and multi-physics (biochemistry, fluid dynamics, structural dynamics) of the tumor micro-environment. Much of the breadth of physiochemical modeling developed for multi-cell biological systems in general is relevant to the present work. Several groups have focused, in particular, on cell deformation and adhesion mechanics [4–14].

In the present work we propose generalized repulsion force and adhesion models to accommodate the local shape of the membrane by accounting for the influence of all discrete membrane faces on all others.

This extends our earlier work [10, 15], and that of others [4] where cell-cell proximity was taken as a single scalar for each cell pair. This generalization is particularly important for bond adhesion kinetics since it allows for bonds to be formed between any two faces, and is inherently consistent with the fully generalized conformal CFD meshing approach adopted, and the ability to model the temporal evolution of many bond pairs in a given cellular interaction. This work also builds upon the Adhesive Dynamics method developed by the Hammer, et al [5, 11, 13, 14]. In this paper, the Adhesive Dynamics algorithm is implemented and tested in a fully generalized, three-dimensional, finite-volume, multiphase CFD solver. This implementation allows local system parameters to be used in solving and coupling the biochemical and fluid interactions instead of global, bulk, or average parameters. Such a platform also allows this work to be expanded upon in a number of ways. First, coupling the biochemistry with CFD and 6DOF motion will allow for the exploration of various cellular adhesion and aggregate formation scenarios. Second, structural mechanics can be reintroduced, as per our earlier work [10], either using an elastic membrane approach or by implementation of a unified continuum mechanics formulation [16]. Third, the system to be solved can be expanded to contain an arbitrary number of arbitrarily shaped bodies. The only limitation on the allowable number of bodies would be the amount of available computational resources. The generalized biochemical model presented in this work can be implemented into any CFD framework (such as those developed by Hoskins [10, 15], Jadheev [17], and others [4]) for increased robustness over currently used biochemical models.

In this study, we model a tumor cell aggregation with a white blood cell in a near wall region under flow conditions that we dealt with in many of our earlier experimental and computational studies [1, 2, 10, 15, 18]. The collision and adhesion interactions between a PMN and melanoma cell are modelled. Here we have used a fixed rigid representation for the PMN, corresponding to an observed shape for the appropriate flow shear rate [10, 18]. Although our earlier work does incorporate a complex nucleus+cytoplasm+membrane PMN structural model [10, 15], the focus of this work is on the generalized interactional modeling between cells in a flowing system, and the demonstration of these models are facilitated by using fixed configurations. A melanoma cell is able to approach and interact with a PMN under full six degree-of-freedom (6DOF) motion. Selectins have been neglected in the present model in order to focus on the mechanics of CAM-1 adhesion.

Commercial grid generation software has been used to create a geometric model and discrete volume mesh of the experimental system, including the TC, PMN, and near wall region of the flow chamber. An in-house developed CFD code was instrumented with the adhesion and repulsion force models in a manner consistent with the underlying Navier-Stokes equation discretization. Specifically, these models are applied locally on discrete cell surface faces, where flow forces due to pressure and shear are also applied.

The paper is organized as follows: First, the models for adhesion and repulsion are presented in some detail, with emphasis on their local, three-dimensional, and time-dependent nature. The models are then verified for their consistency in the context of a generalized discrete CFD model. Lastly, several 3D transient simulations of TC-PMN interactions using the proposed localized biochemical model are presented and discussed.

## Methods

The biochemistry routines contain both an adhesion model, representing the ability of molecules to bond and adhere the cells to each other, and a repulsion model, which represents the microvilli pushing the cells apart and other nonspecific repulsion phenomena [19–21]. These routines make it possible to run computational fluid dynamics software that incorporates three-dimensional interactions of biological cells [22]. For convenience, a brief description of all symbols used throughout this work are provided in Table 1.

**Table 1:** List of Symbols.

6*DOF*	Six degrees of freedom
*A*_*L*_	Surface contact area containing ligands
*a*,*b*	Constants
CFD	Computational Fluid Dynamics
*d*	Separation distance between cell surfaces
*F*_*rep*_	Repulsion force acting on cell
*k*_*b*_	Boltzmann’s constant
$k_{on}^{0}$	Rate of bond formation under equilibrium conditions
*k*_*on*_	Rate of bond formation
$k_{off}^{0}$	Rate of bond breakage under equilibrium conditions
*k*_*off*_	Rate of bond breakage
*n*_*L*_	surface density of ligands
*P*	Probability of bond formation or breakage
PMN	Polymorphonuclear neutrophils
*s*	Bond spring constant
*s*_*ts*_	Bond transition state spring constant
T	Temperature
*t*	Time
*ϵ*	Critical separation distance
*λ*	Equilibrium spring length
*m*	Mass
*I*	Principal moment of Inertia
*x*_*i*_	i-th spatial coordinate
*θ*_*i*_	i-th component of angular displacement vector
*r*_*i*_	i-th component of radius vector
$\hat{n_{i}}$	Unit normal vector of i-th discretized face
$\hat{e_{i}}$	Unit vector along line of action of i-th bond
*δ*_*ij*_	Kronecker delta
*τ*_*ij*_	Viscous shear along the i-th face acting in the direction of the j-th spatial coordinate
*F*_*i*_	Force along i-th spatial coordinate
*T*_*i*_	Torque applied to the centroid about i-th spatial coordinate
*p*	Pressure

### Repulsion

A repulsion force, which is modeled as a non-linear spring force, is applied to the surface of each cell as the melanoma cell approaches the white blood cell to represent several of the repulsive forces observed in the system. Cellular repulsion may be due to a variety of reasons including microvilli pushing, electrostatic repulsion, and steric stabilization [19–21]. As the cells approach within a defined critical distance, they will experience a repulsive force defined as

$$\begin{array}{c} F_{rep}=ad+bd^{3} \end{array}$$ (1)

where *a* and *b* are spring constants determined by empirical observations and *d* is the separation distance between the cells. The values of these two parameters have been initiated to *a* = −110 × 10^−6^ N/m and *b* = 600 × 10^6^ N/m^3^ to maintain a cell surface separation of approximately 0.3 *μ*m; however, these values must be calibrated to match empirical findings through future experimentation. The critical distance used to determine when to apply the repulsive force is based on the average length of microvilli, which is 0.5 *μ*m on a neutrophil [5]. This force is then calculated separately for each individual face of the computational grid lying on the cell membrane. The faces that are within the near-contact region will experience a much greater repulsion force than faces on the trailing edges, which do not significantly interact with the opposite cell.

To calculate repulsion, a simple function first sweeps every face throughout the grid. The mesh faces that correspond to a cell surface are then identified. The coordinates are then recorded for every face on a cell surface. There are now six newly defined lists containing the centroid location of every face on the melanoma cell and PMN, respectively.

Next, a distance calculation is performed between the centroids of every possible combination of faces from cell 1 and cell 2. For a given pair of faces, the distance is calculated using the standard distance equation:

$$\begin{array}{c} d=\sqrt{{\left(x_{2}-x_{1}\right)}^{2}+{\left(y_{2}-y_{1}\right)}^{2}+{\left(z_{2}-z_{1}\right)}^{2}} \end{array}$$ (2)

The critical distance represents the sum of the lengths of microvilli from the two cell types. If the distance has been calculated between two particular faces and those faces are separated by a distance greater than the critical distance, the routine moves on to another pair of faces, taking no further action. If two faces have approached each other within the critical distance, the non-linear spring force defined by Eq 1 will be imposed, acting in the direction opposite to a vector connecting their centroids. For this simulation, the critical distance is initialized as *ϵ* = 1.2 *μm*, but must be experimentally confirmed or modified.

This force is then split into its spatial components, and the components are summed across each face of the melanoma cell for the total repulsive force acting on it from all faces on the PMN within the critical distance. A total sum is also calculated, therefore the six bulk components of force and moment acting on the TC are available to the 6DOF solver. To couple the repulsion model with the fluid mechanics, the forces due to repulsion are treated as body forces acting on the bodies of interest in the flow field. Therefore, the repulsion forces will induce changes in the bodies’ motion, as well as the fluid flow field.

### Adhesion

Based on the second law of thermodynamics and equilibrium conditions for bound and unbound molecules, the association rate, *k*_*on*_, and dissociation rate, *k*_*off*_, of *β*-2 integrins binding to ICAM-1 are defined as [23, 24]

$$\begin{array}{c} k_{on}=k_{on}^{0}n_{L}A_{L}exp(\frac{-s_{ts}{\left(d-\lambda \right)}^{2}}{2k_{b}T}) \end{array}$$ (3)

$$\begin{array}{c} k_{off}=k_{off}^{0}exp(\frac{\left(s-s_{ts}\right){\left(d-\lambda \right)}^{2}}{2k_{b}T}) \end{array}$$ (4)

A localized bond formation probability is calculated for each adhesion molecule on the cancer cell. For the first iteration of the simulation, the biochemistry routine will first sweep through all of the faces in the grid, and save lists containing the centroid coordinates and surface area of the faces on the cancer cell and PMN, separately. It will then calculate the distance from each face on the cancer cell to every face on the PMN. If this distance between two particular faces is less than twice the critical distance *λ*[25], the local rate of bond formation, *k*_*on*_, of the adhesion molecules between those on the cancer cell face to those on the PMN face will be calculated. In calculating the local *k*_*on*_, *A*_*L*_ is the area of the PMN face, *n*_*L*_ is the number density of unbound molecules on that face only, and *d* is the local distance that has just been calculated. In this model, we assume that the adhesion molecules are uniformly distributed across the cell surface. We also use the assumption from Dembo et al, 1988, that the molecules are fixed within the plane of the cell membrane, and not able to diffuse laterally throughout the membrane [26]. Per Simon and Green, 2005, we can assume that our time scales are small enough to ignore any cell-mediated changes in molecule expression [27]. Since each molecule has a much smaller area of potential bond formation, this local *k*_*on*_ must be corrected by some factor such that the average local *k*_*on*_ and the *k*_*on*_ computed using the accepted global method are the same value. To allow for this, after calculating the local value of *k*_*on*_ for all of our cancer cell faces, we will multiply each local *k*_*on*_ by (global *k*_*on*_)/(average local *k*_*on*_).

$$\begin{array}{c} k_{on,global}=\left[\sum_{faces}\left(n_{L}A_{L}\right)\right]k_{on}^{0}exp(\frac{-s_{ts}{\left(d_{centroid}-\lambda \right)}^{2}}{2k_{b}T}) \end{array}$$ (5)

$$\begin{array}{c} k_{on,average}=\frac{\sum_{faces}k_{on}}{\text{number of faces}} \end{array}$$ (6)

$$\begin{array}{c} k_{on,corrected}=k_{on}\frac{k_{on,global}}{k_{on,average}} \end{array}$$ (7)

where *d*_*centroid*_ is the distance between the centroids of the two cells. This correction adjusts the model for use on discretized surfaces.

For every non-zero value of *k*_*on*_, probability of bond formation is calculated using [28]:

$$\begin{array}{c} P=1-exp(-k_{on}\Delta t) \end{array}$$ (8)

A random number between 0 and 1 is then generated, with a bond being formed if the value of the random number is less than *P*. The random number generator makes this model probabilistic. If run multiple times, the model should yield similar but not identical results. This formulation was chosen to account for the inherent randomness of an actual biological system and our inability to deterministically define the behaviors of the molecules with absolute certainty. Pertinent information about the formed bond (the types of molecules, and face information for both the cancer cell face and PMN face that have been bonded) will be recorded.

Once all the potential bonds have been tested for bond formation, forces from each existing bond will be calculated. This force calculation models the bonded molecules as linear springs [5, 15], using:

$$\begin{array}{c} F_{b}=s\left(d-\lambda \right) \end{array}$$ (9)

where the force acts along the vector connecting the centroids of the two bonded faces. The spatial components of each bond force will be calculated, and the total force and torque components will be output to the 6DOF routine. This routine will consider all molecules at every time step by repeating for each type of adhesion molecule on the cancer cell. The list containing information on existing bonds will be written out to a file at the end of the routine. As with repulsion, the forces due to the biochemical adhesion are treated as body forces acting on the bodies of interest; thus, the force will have a measurable effect on body motion and the surrounding fluid flow field.

For every subsequent time step, the routine will first read in the list of existing bonds, and calculate the probability of each of those bonds breaking, based on the new positions of the bonded faces. Since *k*_*off*_ always relates to an individual bond and the length of that bond only, the existing calculation can be used [15]:

$$\begin{array}{c} k_{off}=k_{off}^{0}exp(\frac{\left(s-s_{ts}\right){\left(d-\lambda \right)}^{2}}{2k_{b}T}) \end{array}$$ (10)

where *s* is the linear spring constant of the bond, used to calculate bond force, and $k_{off}^{0}$ is the rate of bond breakage under equilibrium conditions. The same probability model is used as for bond formation, and a random number generator determines whether a bond breaks or remains. Bonds that do not break (i.e., the random number generated was greater than *P* of the bond breaking) are re-saved to the list of existing bonds. From here, the routine repeats as previously described, with *k*_*on*_ calculated for all remaining unbound cancer cell adhesion molecules. It is important to note that bond affinity may be adjusted by varying the values of *s* and *s*_*ts*_, thus changing dissociation constant ($K_{dissociation}=\frac{k_{off}}{k_{on}}$). This ability to vary *K*_*d*_ is beneficial as bond affinities can be adjusted as a way of modeling local or global changes in blood chemistry.

### Physics Coupling

In an effort to model cell-cell interactions, various physics acting on the system must be modeled and coupled. The coupling allows for the sharing of information between the component physical systems, allowing for the resolution of complex interactions of these physical systems.

In the present work, the physics of interest are fluid dynamics, biochemical interactions, and rigid body dynamics. Due to the quasi-steady nature of this problem (as discussed in [10, 15, 29]) the rigid body motion was chosen as the coupling mechanism of these dynamic systems [29].

Rigid body dynamics can be described as an extension of Newton’s second law of motion,

$$\begin{array}{c} \frac{d^{2}x_{i}}{dt^{2}}=\frac{F_{i}}{m} \end{array}$$ (11)

$$\begin{array}{c} \frac{d^{2}\theta_{i}}{dt^{2}}=\frac{T_{i}}{I} \end{array}$$ (12)

where *x*_*i*_ is the linear displacement vector in the *i* ^*th*^ direction, *θ*_*i*_ is the angular displacement vector about an axis parallel to the *i* ^*th*^ principal axis and passing through the body’s centroid, *F*_*i*_ is the sum of all forces applied at the centroid in the *i* ^*th*^ direction, *T*_*i*_ is the torque applied to the centroid about an axis parallel to the *i* ^*th*^ principal axis and passing through the body’s centroid, *m* is the mass of the body, and *I* is the principal moment of inertia corresponding to the *i* ^*th*^ principal axis.

Once the sum of forces and torques acting on a body have been computed, Eqs 11 and 12 can be used to compute the motion of the body. The computed motion is then used to update the position and velocity of the body and update the CFD solver’s boundary conditions. It must be noted that, in the present work, the PMN is assumed to be rigid and fixed to the substrate. Hence, the sum of all forces and torques acting on the PMN are defined to be identically zero, respectively. Additionally, the TC is treated as a rigid sphere with constant and uniform density, allowing for mass and principal moment of inertia to be found using known geometric properties.

For the TC-PMN interaction problem, the motion of the melanoma cell is caused by the forces due to hydrodynamics and the forces of the biochemical interactions. Therefore, the forces due to these physical systems must be computed.

In the context of a discretized surface, the forces and torques due to hydrodynamics acting on a body, given the solution to the flow field, can be computed as

$$\begin{array}{c} F_{fluid}=\int_{A}\left[\left(-p\delta_{ij}+\tau_{ij}\right){\hat{n}}_{j}\right]dA \end{array}$$ (13)

$$\begin{array}{c} T_{fluid}=\int_{A}\left\{r_{fluid}\times \left[\left(-p\delta_{ij}+\tau_{ij}\right){\hat{n}}_{j}\right]\right\}dA \end{array}$$ (14)

where ${\hat{n}}_{j}$ is the unit normal of the *j* ^*th*^ face on the body, *r*_*fluid*_ is the radius vector from face *j* to the centroid of the body, and *δ*_*ij*_ is the Kronecker delta.

Similarly, the forces and torques due to the biochemical adhesion can be found in the context of a discretized surface. Approximating bonds to behave as Hookean springs allows the forces and torques action on the body to be expressed as

$$\begin{array}{c} f_{bond}=s\left(d-\lambda \right){\hat{e}}_{j} \end{array}$$ (15)

$$\begin{array}{c} F_{bond}=\sum_{bonds}[\left(f_{bond}•{\hat{n}}_{i}\right){\hat{n}}_{i}] \end{array}$$ (16)

$$\begin{array}{c} T_{bond}=\sum_{bonds}\{r_{bond}\times [\left(f_{bond}•{\hat{n}}_{i}\right){\hat{n}}_{i}]\} \end{array}$$ (17)

where *s* is the spring constant, *d* is the distance between the two molecules, ${\hat{n}}_{i}$ is the unit normal along the *i* ^*th*^ coordinate axis, *r*_*bond*_ is the radius vector from face containing the *j* ^*th*^ bond to the centroid of the body, and ${\hat{e}}_{j}$ is the unit normal in the along the line of action of the *j* ^*th*^ bond.

Lastly, the forces and torques due to the cellular repulsion must be computed. Using Eq 1, we can express the repulsion as

$$\begin{array}{c} f_{rep}=\left[ad+bd^{3}\right]\hat{e} \end{array}$$ (18)

$$\begin{array}{c} d=\sqrt{{\left(x_{2}-x_{1}\right)}^{2}+{\left(y_{2}-y_{1}\right)}^{2}+{\left(z_{2}-z_{1}\right)}^{2}} \end{array}$$ (19)

$$\begin{array}{c} F_{rep}=\sum_{faces}f_{rep} \end{array}$$ (20)

$$\begin{array}{c} T_{rep}=\sum_{faces}\{r_{rep}\times f_{rep}\} \end{array}$$ (21)

where *a* and *b* are spring constants representing the forces due to the various repulsive phenomena previously mentioned, *d* is the distance function evaluated between the two faces, *r*_*rep*_ is the radius vector from a computational face to the centroid of the its body, and $\hat{e}$ is the unit normal vector along the line of action between the two computational faces. For the TC-PMN cell pairs being simulated in this paper, the spring constants have been set to *a* = −110 × 10^−6^N/m and *b* = 600 × 10^6^N/m^3^ to maintain a minimum separation of 0.3 *μ*m. In this work, the repulsive force is calculated and applied at separation distances less than *ϵ* = 1.2*μ*m.

Therefore, the total sum of forces and torques acting at the body centroid are computed as

$$\begin{array}{c} F=F_{fluid}+F_{bonds}+F_{rep} \end{array}$$ (22)

$$\begin{array}{c} T=T_{fluid}+T_{bonds}+T_{rep} \end{array}$$ (23)

These vectors can then be split into components and used to solve Eqs 11 and 12.

### Computational Verification

MATLAB codes of the adhesion calculations were created during the development of the adhesion model. MATLAB was used before the initial implementation of the adhesion model to simulate the intended calculations without the presence of a mesh or fluid. This was used to ensure the continuity of variable definitions, initializations and agreements. MATLAB was later used to isolate the activity of the adhesion model and verify proper behavior within NPHASE, an in-house developed CFD code used in previous work [10].

**Fig 2:**
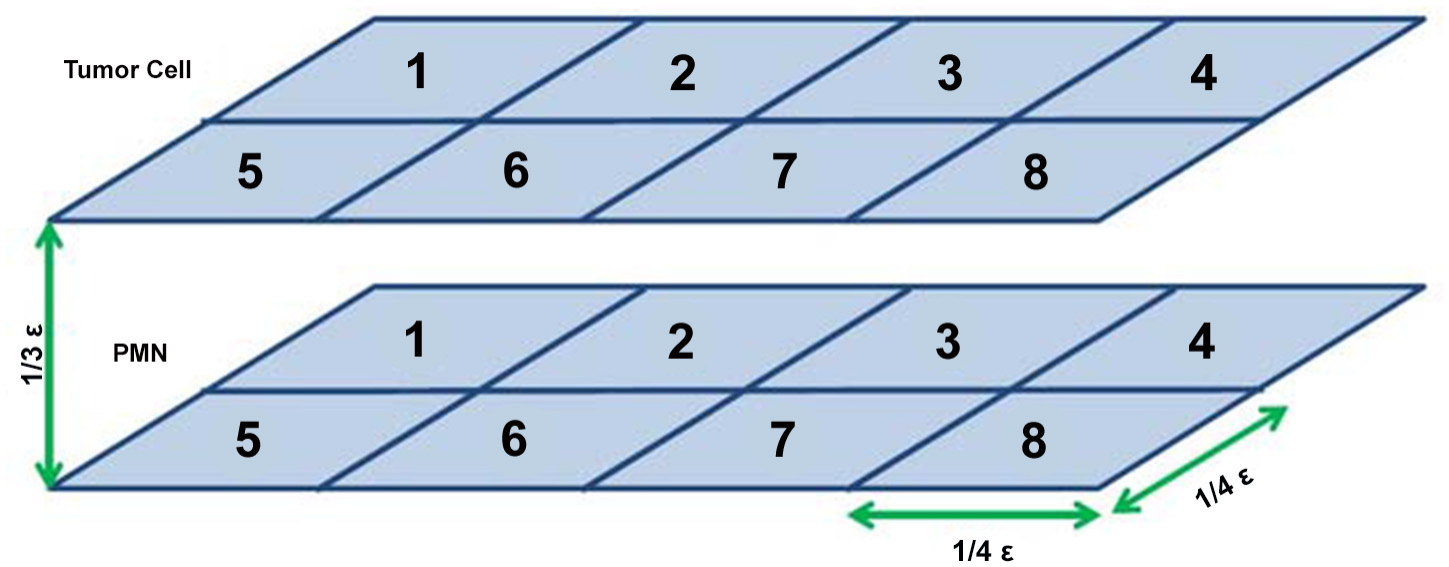
Simplified computational mesh used for model validation. This simplified version of a mesh was used to run the Adhesion model through MATLAB without requiring the input of the detailed geometric mesh used by NPHASE.

To test the ability of the adhesion model to calculate distances between faces, two lists, one representing each cell, were created containing eight centroid locations for parallel rectangular planar surfaces, as shown in Fig 2. Based on the molecule density and the face area, the code calculated the number of molecules that would be on each face. It then calculated the distance between every possible pair of faces, and generated an 8x8 matrix of distance values. For any distance less than the critical adhesion distance, *ϵ*, a value of *k*_*on*_ was calculated as well. The values of *k*_*on*_ are also saved in an 8x8 matrix, and if the two faces corresponding to a position in this matrix are at a greater distance from each other than the critical adhesion distance, that *k*_*on*_ value is saved as zero. Every time a non-zero value for *k*_*on*_ is calculated, it is added to a sum of all *k*_*on*_ values calculated, so that the average can be compared to a “global” contact area wide value for *k*_*on*_. The ratio of the average local *k*_*on*_ and the contact area global *k*_*on*_ is used to correct each local value, so that the global value becomes the average local value.

The next step of building the MATLAB model was to generate bonds. Rather than using the probability function with a random number generator, this phase of the model was meant to verify what molecules were being allowed to bond. Therefore, if a value of *k*_*on*_ was calculated (that is, if the two faces were within the critical adhesion distance from each other), the probability of bond formation was set as *P* = 1, or in other words, there is a 100% chance that a bond will form for every available molecule on that face. This was done in order to determine whether the calculated number of molecules per face was actually limiting the number of bonds that could form. The initial formulation of the adhesion model allowed the molecules on the PMN to be unlimited, and the number of molecules would impact the rate of bond formation but would not prevent more bonds forming than there were molecules available. To correct this, a new list was created to keep track of the number of unbound molecules on the PMN faces, and this value was used to modify the *k*_*on*_ value of ICAM-1 as more bonds were formed.

The same issue needed to be addressed on the melanoma cell. Since the PMN is currently allowed to express two adhesion molecules (LFA-1 and Mac-1), the algorithm must ensure that any individual molecule on the melanoma cell would form multiple bonds at a single point in time. Based on the current formulation of the routine, including the modification to the PMN adhesion molecules made in the previous step, every molecule on either cell will be used no more than once *per molecule-type pair*. Because ICAM-1 molecules are considered twice, and given the ability to interact with either LFA-1 or Mac-1, each one of them had the ability to form up to two bonds.

To address this issue, the entire routine was encompassed within two overall loops, where previously there had been one. When the routine was first built, a loop with one iteration per molecule-pair-type controlled the entire adhesion model and the biochemistry calculations were repeated for the possible molecule pairings. In this simulation, there were two iterations of the loop, one for ICAM-1 to LFA-1 bonding and one for ICAM-1 to Mac-1 bonding. There will now be two loops, one that has an iteration for every molecule on the tumor cell (in this case, there is only one, but the model needs to be robust enough to accommodate for a different cell type that is defined as having multiple relevant adhesion molecules), and one that has an iteration for every molecule on the PMN (in this case, there are two).

The previous step laid the groundwork for the model to accommodate for a tumor cell with any number of molecule types on the surface, rather than the current model, which has only one. However, if a second molecule type were present on the tumor cell, it would allow for the same issue of double-counting molecules that was just corrected for the PMN adhesion molecules.

To correct this problem, and complete the robustness of the routine to be able to accurately calculate the interactions between any number of molecule types, the lists that were already defined to track available molecules on each face were modified to contain two values. Two separate indexes, one representing the face on the cell surface (as before) and one representing the molecule type, determine the position within the 2-dimensional list where information regarding available molecules is stored. Every time a bond forms, one available molecule is subtracted from each of the involved faces. With these 2-dimensional lists, controlled by the overall loops of the routine, the information regarding unbound molecules will remain saved throughout all iterations of molecule types and face references.

Based on the existing values for parameters, cell surfaces will never get close enough for bonds to form. The length of microvilli, which dictates how close cell surfaces can approach each other before being inhibited by repulsion forces, is orders of magnitude greater than the length of adhesion molecules, which dictates how close cell surfaces must approach each other before bonding will be considered. To correct for this influence and allow for the propagation of bond formation between the two cells, the critical adhesion distance is modified to be the actual critical adhesion distance plus the critical repulsion distance. This formulation assumes that all adhesion molecules at all locations across the cell surface are located at the fully-extended tips of microvilli. Experimental verification will be required to determine whether this formulation, although physically inaccurate, is representative of the actual behavior of adhesion molecules. Otherwise, some other modification may be required to allow for binding between the two cells.

## Results

In this study, we model the collision and adhesion interactions between a PMN and melanoma cell in the context of fully 3D CFD simulations. In this section, we discuss results obtained from implementing the proposed models into a 3D CFD solver using representative geometries of PMN and melanoma cells [18].

### Model Problem and Computational Implementation

As mentioned earlier, this study models a tumor cell aggregation with a white blood cell in a near wall region under specified flow conditions. In a general sense, this problem is approached as two rigid bodies in a flow field. In this approach, the no-slip condition on the surface of these rigid bodies impose boundary conditions on the flow problem. The no-slip condition on the bodies’ surfaces, along with the inflow, outflow, and domain wall boundary conditions allow for a problem well-suited to be solved using CFD tools. Using these boundary conditions, the steady, incompressible Navier-Stokes and continuity equations governing the flow system are solved using NPHASE-PSU, an in-house developed finite-volume CFD code using a segregated pressure based methodology [30].

The nature of the localized biochemisty model proposed in this work allows the model to be implemented as a subroutine within NPHASE-PSU, replacing the bulk biochemistry model used in our previous work [10]. Whereas the bulk biochemistry model uses global parameters to determine the probability of bond formation and breakage between two bodies of interest, the proposed model computes probability of bond formation and breakage for every possible bond using local parameters and applies an appropriate correction factor to ensure the results are not dependent on the computational setup. The biochemistry equations are solved once the flow field has been obtained, which allows for the exploitation of the existing data structures used for the CFD solution procedure. Once the flow and biochemistry equations have been solved, it is possible to compute the values of *F*_*i*,*fluid*_, *F*_*i*,*bonds*_, *F*_*i*,*rep*_, *T*_*i*,*fluid*_, *T*_*i*,*bonds*_, and *T*_*i*,*rep*_.

It must also be noted that, while this work is focused on the described model problem, the generalized biochemistry model presented can be implemented in any computational tool used to model biochemical interactions of neighboring bodies. Another system where this generalized biochemistry problem may be appropriate is selectin-mediated leukocyte rolling as presented by Jadav, et al [17]. In the leukocyte rolling system, our generalized biochemistry model would compute probabilities of bond formation for every bond physically possible, as well as computing the forces due to various repulsive phenomena. This capability would allow the tool in [17] to not only model each individual bond, but also account for the repulsive nature of cellular interactions.

### Repulsion

The addition of the repulsion force allows for the melanoma cell to flow smoothly when near the PMN. By modeling the cells as smooth rigid bodies, the simulation is not able to resolve the repulsive effects of the microvilli on the cell surfaces. Without the imposed repulsion force, the cells become non-physically close. As the cells collide, infinitesimally small time steps are required to resolve the hydrodynamic forces in the near-collision region of the flow. These small time steps severely increase simulation runtime, while a non-physical collision invalidates the results.

**Fig 3:**
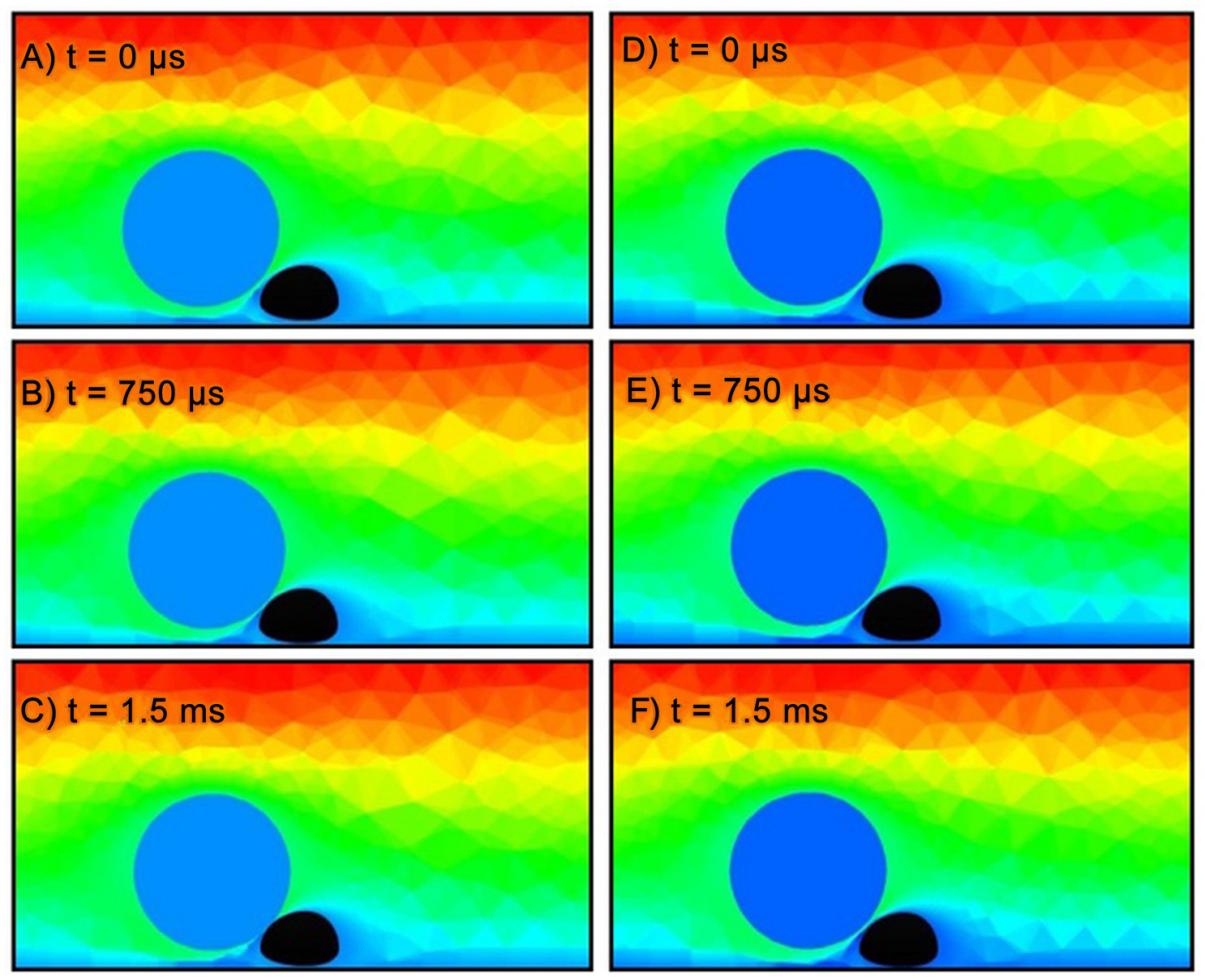
Comparison of CFD results using same initial parameters, with and without repulsion. (a-c) show results for the CFD simulation without the cellular repulsion. Without repulsion, the bodies can collide and cause the CFD solver to crash. When a collision occurs, as shown in (c), the CFD may produce non-physical results or be unable to reach a converged solution. (d-f) show results for CFD simulation with cellular repulsion activated. In this case, the bodies do not collide and the simulation continues to advance in time. *Note: results are 2D cross-sections of fully 3D simulations. Flow from left to right. Contours are normalized velocity magnitude (blue = 0, red = 1). PMN is colored in black and melanoma cell is colored in blue.*

**Fig 4:**
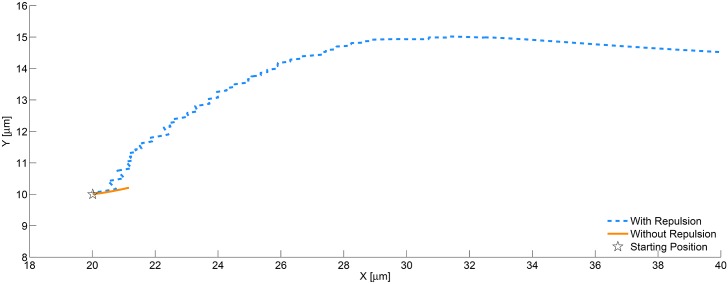
Trajectories of melanoma cell from CFD simulation, with and without repulsion. The results from the simulation shown in Fig 3 is post-processed to extract the centroid location of the melanoma cell throughout the simulation. That data was used to create a trajectory for each of the two cases. The case without repulsion (solid line) has a short path due to the collision with the PMN cell. The case with repulsion activated (dashed line) shows the body moving over and pass the PMN body. In this particular simulation, the motion of the centroid is planar (z = constant).

For sake of direct comparison, two simulations were run to observe the effects of the repulsion force. The two simulations were initialized with the same input parameters, found in Table 2. In both cases the melanoma cell was intentionally initialized in close vicinity of the white blood cell, so that the effects of the repulsion force would have immediate impact. The first case was run with only hydrodynamic forces affecting cell trajectory. As shown in Fig 3c, the melanoma cell in the first case collides into the PMN and remains there for the duration of the simulation. The second case uses both hydrodynamic and repulsion forces in the 6DOF motion computation. Fig 3(d-f) shows the repulsion model prevents the bodies from colliding. Fig 4 shows the melanoma cell in this case having a smooth trajectory over and away from the PMN, thus ensuring the repulsion model captures the repulsive effects of the true cell surface geometry. The trajectories shown in 4 are calculated using the 6DOF motion algorithm described in [29].

**Table 2:** Simulation Initial Parameters.

Parameter	Value
Domain Size [*μ*m]:	
X	60
Y	32
Z	42
Tumor Cell Centroid [*μ*m]:	
X	20
Y	10
Z	21
PMN Centroid [*μ*m]:	
X	30
Y	2.5
Z	21
Timesteps	100

### Adhesion

**Fig 5:**
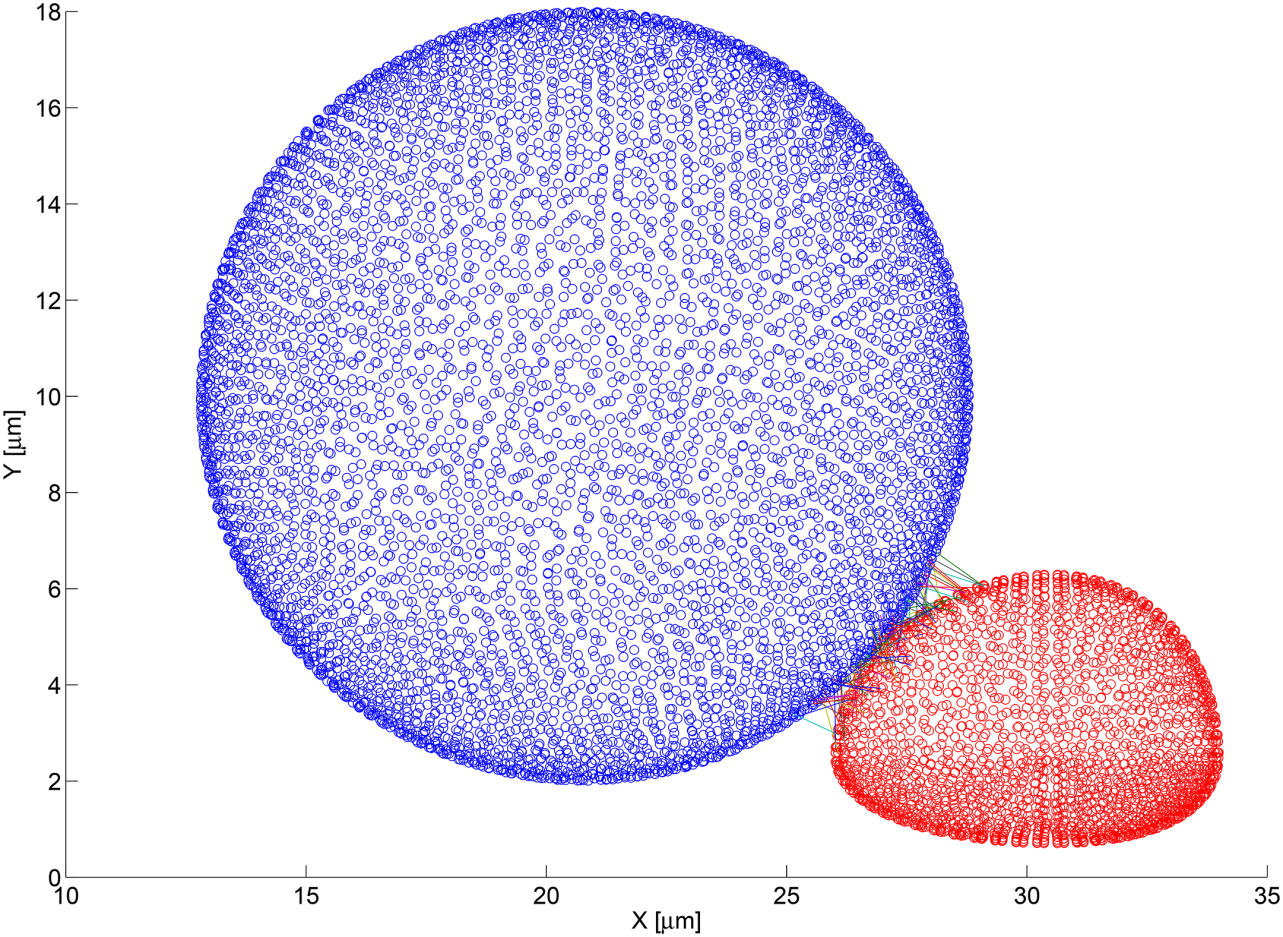
Bond formation between nearby cells using the fine computational mesh. The blue figure is a 3D point cloud of the fine computational mesh used to describe the surface of the melanoma cell. Each blue circle represents the centroid of a discretized mesh face. Similarly, the red figure is a 3D point cloud of the fine computational mesh used to describe the surface of the PMN. The lines connecting the circles represents bonds that have formed and connect the two faces on which the involved adhesion molecules reside.

By initializing the tumor cell in close proximity to the neutrophil, it is possible to verify bond formation without running a full CFD simulation, and only considering a single instance in time. By saving the locations of all pairs of bound faces, Fig 5 was generated. In this simulation, the case was initialized to the values found in Tables 2, 3, and 4.

**Table 3:** Adhesion Molecule Surface Density.

	Molecule	Surface Density	Reference
Melanoma Cell:	ICAM-1	13 x 10^12^ molecules / *m* ^2^	Simon and Green, 2005 [27]
PMN:	LFA-1	45 x 10^12^ molecules / *m* ^2^	Simon and Green, 2005 [27]
	Mac-1	5 x 10^12^ molecules / *m* ^2^	Simon and Green, 2005 [27]

**Table 4:** Adhesion Parameters.

	LFA-1 to ICAM-1	Mac-1 to ICAM-1	Reference
*k*_*on*_	3000 1/Ms	3000 1/Ms	Hoskins, 2008 [15]
*k*_*off*_	0.3 1/s	0.29 1/s	Hoskins, 2008 [15]
s	2 × 10^−3^ N/m	2 × 10^−3^ N/m	Hammer and Apte, 1992 [5]
*s*_*ts*_	1 × 10^−3^ N/m	1 × 10^−3^ N/m	Hammer and Apte, 1992 [5]
*λ*	0.05 *μ*m	0.05 *μ*m	Springer, 1990 [25]

To ensure the results of the biochemical adhesion model were not dependent on the CFD mesh resolution, the CFD simulation was run for one instance in time with identical boundary conditions but differing mesh resolutions; a coarse mesh with a maximum element size of 1.5 *μ*m and a fine mesh with a maximum element size of 0.5 *μ*m were used to for the mesh dependency analysis.

**Fig 6:**
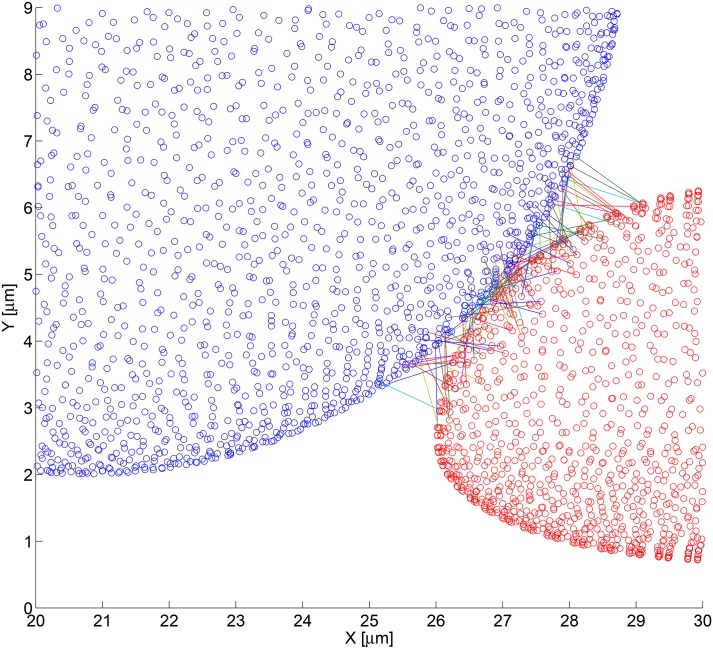
Zoomed view of bond formation using the fine computational mesh. This is a zoomed view of the bond formation and 3D point clouds of the PMN and melanoma cell using the fine mesh (shown in Fig 5). A total of 96 bonds were formed and are shown. Note: multiple bonds may occur between the same face pairs.

**Fig 7:**
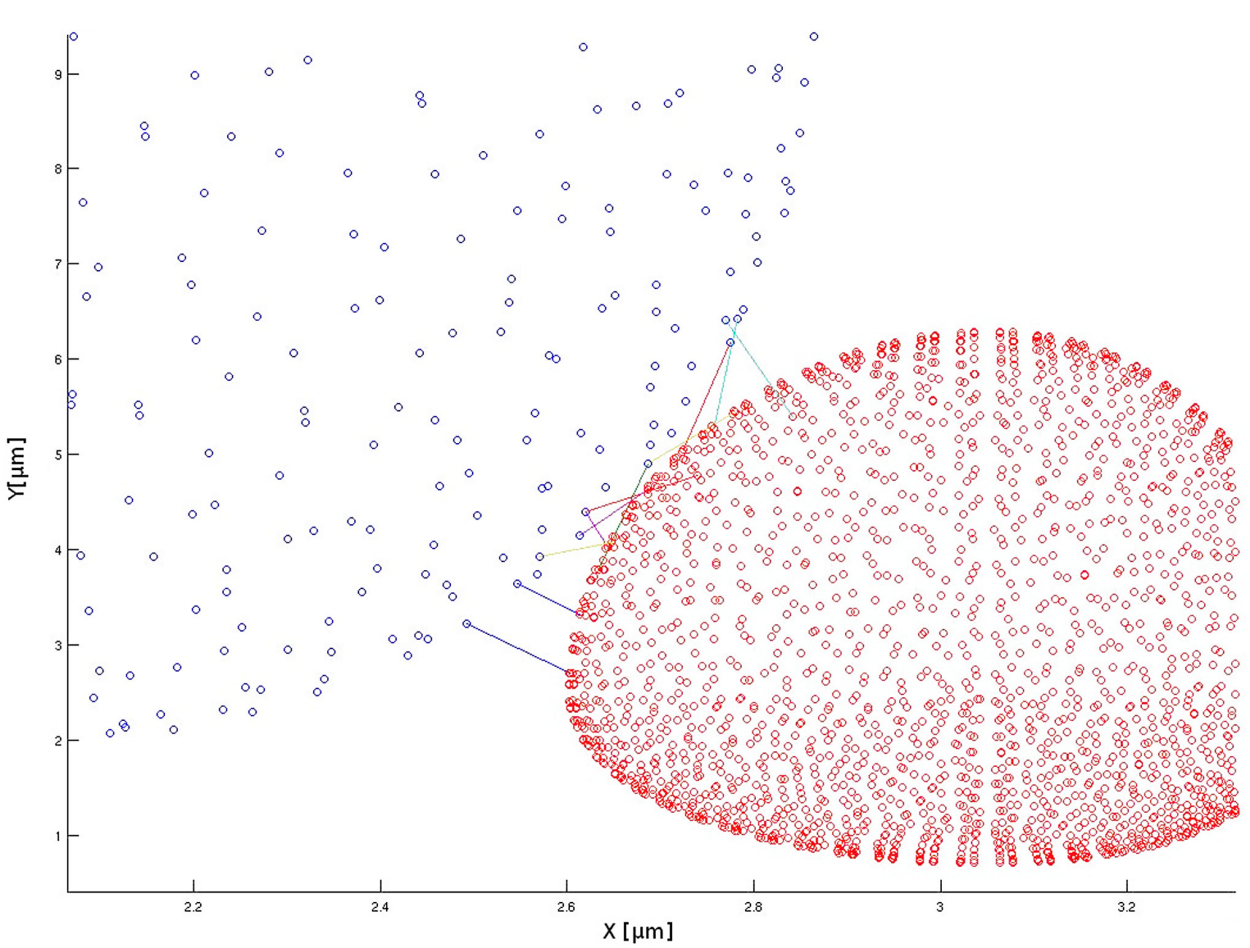
Zoomed view of bond formation using the coarse computational mesh. This is a zoomed view of the bond formation and 3D point clouds of the PMN and melanoma cell using the coarse mesh. With the coarse computational mesh, 97 bonds were formed and are shown. Each face pair contains multiple adhesive bonds, which allows for an appropriate number interactions although fewer computational faces are involved in the calculations. Note: multiple bonds may occur between the same face pairs.

The simulation that generated the bond distribution seen in Fig 6 used a tumor cell discretized with the fine mesh parameters. Every blue circle in the figure represents the centroid of a tumor cell mesh face. A total of 96 bonds were generated between the two cells. To verify whether bonding is dependent on the mesh sizing, the simulation was rerun using the coarse mesh as shown in Fig 7. Although there are fewer pairs of faces involved in the bond calculations, the subgrid biochemistry model accounted for this difference and yielded a total of 97 bonds were formed. It can be assumed that the slight variation is due to the stochastic nature of the adhesion routine.

## Discussion

### Repulsion

As previously stated, there are three parameters within this model that have been assigned values that need verification. Experimental data should be provided to confirm the values of a, b and *ϵ*.

Because the cell cannot currently deform, forces that actually would be acting on a local area of the cell surface result in a net movement of the cell. These repulsive forces are more likely to force the contacting faces to flatten, rather than the entire cell to alter its path. It is likely that if the cell surfaces could deform, the localized retraction of an area of the cell surface would affect the average separation distance between the two cells and the calibration of the constants.

### Adhesion

The first design challenge of this model was to assign a location to every simulated molecule. The assumption first had to be made that adhesion molecules are evenly distributed across the cell surface. Although this may be unlikely, as the molecules are free to move throughout the membrane. Empirical data that suggests a non-uniform distribution that could be replicated in this model was not found in the literature. Furthermore, for the purposes of our model, adhesion molecules outside of the contact area between the two cells are neglected. Therefore, it is only necessary that adhesion molecules be uniformly distributed within the contact area for our model to be spatially accurate. As previously stated, our ability to assign locations of molecules in space within the model is dictated by the resolution of the cell surfaces within the discretized computational grid. Adhesion molecules are assigned a location based on the number density of molecules across the cell surface area and the area of an individual face on the cell surface mesh. Every molecule on a given face is assumed to be located at the centroid of that face, and so the spatial uniformity of the molecules is dictated by the refinement of the mesh. As a consequence of this mesh dependency, there may be a non-integer number of molecules on a given face to ensure the proper number of globally available molecules. If a rounding method had been used there would be no way to maintain the uniformity of molecules across the surface. The loss of uniformity would impart some inherent bias to areas that had been rounded up rather than rounded down. This bias would make it very difficult to maintain the proper overall number density, and ensure that a proportional number of molecules had been either rounded up or down such that the total number of molecules on the surface was representative of a real cell. To accommodate for the non-integer number of molecules, the loop controlling the random number generator was modified such that it would calculate a random number for every molecule or fractional molecule on the face. For each full molecule, the random number is compared against the probability value that was found for that face. For the fractional molecule, the random number is compared against a modified probability, which is the original probability value multiplied by the fractional amount of the molecule. For instance, if a given face is found to have 8.3 adhesion molecules on its surface, nine random numbers will be generated. The first eight will be compared against the probability value *P*, and the ninth will be compared against 0.3*P*, thus making it less likely but still possible for a bond to form.

Next was the assessment of the probability model controlling bond breakage kinetics. In general, it is assumed that a given bond has a certain expected lifetime, which makes it more likely for the bond to break after it has existed for a given period of time. This would require each formed bond to have an indicator of how many timesteps the bond has existed. However, in general these calculations are only done across the population, and represent the total number of bonds that exist at a certain time. Since the probability of bond breaking is tested at every timestep, each probability test can be assumed to represent an independent event. These independent events are all dictated by the same probability of breakage and consider only the amount of time that has elapsed since bond breakage was last calculated. The increased likelihood of bond breakage is accommodated for by allowing the bond multiple opportunities to break.

Having bonds exist through multiple timesteps means their location in space must be recorded. In a single timestep, the location of a bond is defined by the location of the two interacting mesh faces, and is easily stored within NPHASE. However, the grid is regenerated every timestep.

The final consideration was how to calculate *k*_*on*_ in a local setting in a way that both takes advantage of the CFD capabilities and remains aligned with empirical data and models from real cells. No model currently exists representing a distribution of bond formation rates across a cellular contact area. As has been previously discussed, the model used here assumes that the existing model for calculating *k*_*on*_,

$$\begin{array}{c} k_{on}=A_{L}\left(n_{L}\right)k_{on}^{0}exp(\frac{-s_{ts}{\left(d-\lambda \right)}^{2}}{2k_{b}T}) \end{array}$$ (24)

can be scaled down to represent individual grid face areas and distances while maintaining the accuracy of the model. The values of *k*_*on*_ given by this equation will be proportional to their actual distribution, and must be adjusted by a global *k*_*on*_ value such that the average local value and the global value are equivalent. This theory for finding a non-uniform local *k*_*on*_ distribution is based exclusively on the assumption that the existing model is accurate at the molecular (rather than just cellular) scale.

According to the adhesion model equations used here, each molecule has the ability to behave as a spring. The value of *s*_*ts*_ represents the ability of a molecule to deform from its equilibrium length and form bonds with other molecules within a certain range of distances centered around the equilibrium distance [5]. The computational routine forces the value of *k*_*on*_ to zero when the distance is greater than double the equilibrium distance. Because the equations are only meant to mimic molecular behavior and are only approximations, a value of *k*_*on*_ could be calculated at every distance. The value would become prohibitively small for bond formation at distances much greater than the equilibrium distance. However, distances only slightly deviating from equilibrium, *k*_*on*_ would be non-zero and bonding would be possible. The cut-off distance is used to limit unnecessary calculations.

Currently, the cells in the simulation are modeled as non-deformable bodies. Previous work has shown that the ability to deform will play a critical role in the binding kinetics between the two cells [6, 8]. It is expected that there would be less bond formation in the simulated rigid cells than in real cells, given the same rate of bond formation. Therefore, altering the parameters used to calculate *k*_*on*_ within the adhesion model such that the overall total bonding observed and modeled are equivalent would likely lead to making each simulated molecule more likely to form a bond than any real molecule. Calibration of this part of the model must wait until cell deformation is incorporated. The model is generalized so that adhesion can be calculated using the same method regardless of the geometry of the cells, since the routine accepts the location of each face in the grid as input. Altering the deformability of cells will only change the values of input to this routine, and will have no effect on how that input is processed. The authors are pursuing the reintroduction of structural mechanics modeling as demonstrated in our earlier work [10, 15], and that advancement will not require modification to the biochemical interaction model.

Many researchers have determined methods for empirically calculating the spring constant, *s*, of a molecule [31–33]. Bell et al. (1984) determined numerically that the spring constant of *α*-helical proteins must fall between 10^−2^ and 10^3^ dyn/cm, and their approximation of *s* = 0.1 dyn/cm has since been largely accepted [20]. Recent researchers have used atomic force microscopy to apply a force to opposite ends of a bound molecular complex and determine the magnitude of the applied force, as well as measure the change in length of the complex in order to determine an empirical value *s* representing the linear spring constant [33, 34].

Experimental data so far has suggested that the interactions between ICAM-1 and LFA-1 are primarily responsible for the initial tethering of a melanoma cell to a PMN [1]. The interactions between ICAM-1 and Mac-1 played a greater role in stabalizing the adhesion between the two cells, even in the presence of shear flow, after the initial tethering had taken place. It is likely that this difference of roles between the two molecule types should be reflected in the simulation by different parameters describing their behaviors. Possibly the initial reliance on LFA-1 can be explained simply by its increased membrane concentration compared to Mac-1. However, the distinction of Mac-1 playing a greater role in combating shear forces possibly reflects a difference in the strength of bonds formed between ICAM-1 and Mac-1, or a difference in the typical lifespan of those bonds as reflected in $k_{off}^{0}$. The bond strength variation, if proven to be the case, should be reflected in a different value for the spring constants that represent molecular bond forces as spring forces for the ICAM-1 to Mac-1 interaction than the value assigned to the ICAM-1 to LFA-1 interaction. Currently both types of bonds are governed by the same parameters, but it is likely that experimental verification of parameter values will reveal differences between the two molecule types that can explain their differences in behavior.

### Conclusions and Future Work

The major contribution of this work has been the development of a numerical model for computing local bond formation rates in a manner that is consistent with a time dependent Computational Fluid Dynamics (CFD) framework of a full system model with an arbitrary number of interacting flowing cells.

The assumption upon which this model was derived is that the existing calculation for *k*_*on*_ at the cellular-level is physically accurate and therefore can be extrapolated to a smaller scale. The equation itself implies that bonding is most likely to occur at exactly the equilibrium distance, and becomes less likely if the molecules are either too close to each other or if they are further away.

The model was presented in detail, verified to behave as required in multi-cell, multi-face simulations, and has been implemented for a flowing TC-adherent PMN system. It was also found that the model is not dependent of the resolution of the CFD mesh, which allows mesh resolution to be solely determined by the fluid dynamics.

As this capability has been established, the authors are pursuing several advancements including: 1) A multiscale simulation effort, wherein the cell-molecular scale presented here is interfaced with population scale modeling to predict clinical environment aggregation behavior, 2) At the molecular level, calibration of parameters for $k_{on}^{0}$ and $k_{off}^{0}$ using comparisons with bulk adhesion observations in the literature and our laboratory, 3) Inclusion of many different cell-type combinations, not only the TC-PMN pair, and, 4) Reintroduction of structural mechanics modeling per our earlier work [10], 5) Incorporation of a physically-based localized repulsion model to resolve nonspecific repulsive forces.
